# Are medical history data fit for risk stratification of patients with chest pain in emergency care? Comparing data collected from patients using computerized history taking with data documented by physicians in the electronic health record in the CLEOS-CPDS prospective cohort study

**DOI:** 10.1093/jamia/ocae110

**Published:** 2024-05-23

**Authors:** Helge Brandberg, Carl Johan Sundberg, Jonas Spaak, Sabine Koch, Thomas Kahan

**Affiliations:** Division of Cardiovascular Medicine, Department of Clinical Sciences, Danderyd Hospital, Karolinska Institutet, Stockholm SE-182 88, Sweden; Department of Learning, Informatics, Management and Ethics, Karolinska Institutet, Stockholm SE-171 77, Sweden; Department of Physiology & Pharmacology, Karolinska Institutet, Stockholm SE-171 77, Sweden; Division of Cardiovascular Medicine, Department of Clinical Sciences, Danderyd Hospital, Karolinska Institutet, Stockholm SE-182 88, Sweden; Department of Learning, Informatics, Management and Ethics, Karolinska Institutet, Stockholm SE-171 77, Sweden; Division of Cardiovascular Medicine, Department of Clinical Sciences, Danderyd Hospital, Karolinska Institutet, Stockholm SE-182 88, Sweden

**Keywords:** chest pain, medical history taking, artificial intelligence, acute coronary syndrome, medical informatics

## Abstract

**Objective:**

In acute chest pain management, risk stratification tools, including medical history, are recommended. We compared the fraction of patients with sufficient clinical data obtained using computerized history taking software (CHT) versus physician-acquired medical history to calculate established risk scores and assessed the patient-by-patient agreement between these 2 ways of obtaining medical history information.

**Materials and methods:**

This was a prospective cohort study of clinically stable patients aged ≥ 18 years presenting to the emergency department (ED) at Danderyd University Hospital (Stockholm, Sweden) in 2017-2019 with acute chest pain and non-diagnostic ECG and serum markers. Medical histories were self-reported using CHT on a tablet. Observations on discrete variables in the risk scores were extracted from electronic health records (EHR) and the CHT database. The patient-by-patient agreement was described by Cohen’s kappa statistics.

**Results:**

Of the total 1000 patients included (mean age 55.3 ± 17.4 years; 46% women), HEART score, EDACS, and T-MACS could be calculated in 75%, 74%, and 83% by CHT and in 31%, 10%, and 25% by EHR, respectively. The agreement between CHT and EHR was slight to moderate (kappa 0.19-0.70) for chest pain characteristics and moderate to almost perfect (kappa 0.55-0.91) for risk factors.

**Conclusions:**

CHT can acquire and document data for chest pain risk stratification in most ED patients using established risk scores, achieving this goal for a substantially larger number of patients, as compared to EHR data. The agreement between CHT and physician-acquired history taking is high for traditional risk factors and lower for chest pain characteristics.

**Clinical trial registration:**

ClinicalTrials.gov NCT03439449

## Graphical Abstract

**Figure:**
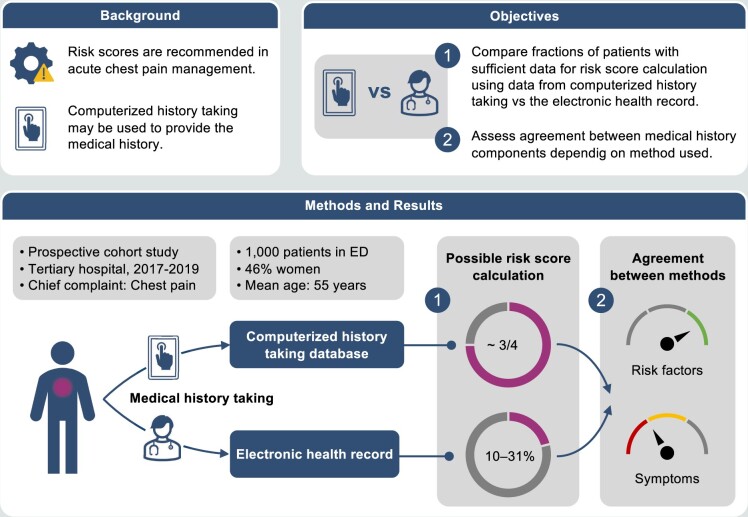

## Background and significance

Chest pain is a common complaint in patients attending emergency departments (EDs) globally.^1–3^ Accurate and timely diagnosis of the minority of these patients with life-threatening illness, such as an acute coronary syndrome (ACS), is critical.^4^ At the same time, it is important to identify patients with low-risk chest pain, who can be discharged safely and relatively quickly in order to reduce the risks and costs of over-testing and the burden on EDs.^5^^,^^6^ Risk stratification tools that integrate medical history data (symptoms and risk factors), ECG findings, and levels of circulating biomarkers are recommended as aids in these regards.^7^ Clinical studies have established the value of several of these,^8–10^ including History, ECG, Age, Risk factors and Troponin (HEART) score,^11^ ED Assessment of Chest Pain Score (EDACS),^12^ and Troponin-only, Manchester Acute Coronary Syndrome (T-MACS).^13^ It appears, however, that physicians overestimate their ability to predict accurately patient-specific risk for ACS in patients with chest pain^14^ and that they underutilize risk stratification tools for chest pain.^15^

Computerized history taking (CHT), in which patients interact directly with expert system software to respond to questions posed by a computer emulating a physician, can provide more complete and accurate medical history data, as compared to history taking by physicians.^16–20^ We and others have shown that CHT is well-accepted by patients.^21–23^ We have also shown that CHT can acquire structured medical history data for risk stratification by the HEART score from most ED patients with acute chest pain.^24^ However, prevailing evidence for risk stratification with risk scores is based on the input of medical history data collected by physicians.^11–13^ Thus, a comparison of data acquired by CHT and by physician-acquired history taking is warranted.

## Objective

This study aimed to compare the fraction of patients with acute chest pain and sufficient data obtained by CHT or by traditional physician-acquired medical history to calculate 3 established risk scores for acute chest pain in the ED setting to indicate the need for hospitalization or to allow for a safe disposition to home from the ED. Second, we assessed the agreement between specific medical history data items collected by CHT and those entered in EHR by traditional physician-acquired history taking.

## Methods

### Study design and setting

The Clinical Expert Operating System Chest Pain Danderyd Study (CLEOS-CPDS; ClinicalTrials.gov identifier: NCT03439449) is a prospective cohort study with the overall aim to determine the value of CHT for the management of acute chest pain,^25^ which recruited patients presenting at the ED at Danderyd University Hospital (Stockholm, Sweden). As compared to national average in Sweden, Region Stockholm has a slightly younger population (mean age 40.2 vs 41.9 years), a higher proportion of individuals in the working age group (67% vs 64%), a nearly identical gender distribution (49.9% vs 49.7% women), and a higher proportion of foreign-born residents (27.6% vs 20.6%).^26^ Additionally, Region Stockholm exhibits a higher average income compared to the national average (SEK 431 139 vs SEK 367 354, approximately USD 37 400 and USD 31 900), and also a greater income disparity, as indicated by its higher Gini coefficient (0.405 vs 0.361).^27^ All patients with acute chest pain in this ED are triaged by a cardiology consultant or senior cardiology resident (from 08:00 to 17:00) or by a triage nurse (from 17:00 to 08:00) to assess the degree of urgency using the RETTS protocol, as described below.^28^ Following triage, patients are directed either to the ED cardiology unit, with 24-hour service, and staffed by cardiology consultants or senior cardiology residents with limited access to non-invasive cardiac imaging techniques to detect myocardial ischemia, or to the cardiology inpatient day-care unit, operational from 8:00 to 17:00. The day-care unit serves as an observational day-ward, staffed by cardiology consultants and offering easy access to both invasive and non-invasive cardiac imaging techniques. Before admission to the ED cardiology unit or the inpatient day-care unit, ECG and biomarkers are collected. In the ED cardiology unit or inpatient day-care unit, a more thorough examination and standard history taking is performed by the attending physician. The exact disposition of patients has been presented in detail previously.^24^^,^^25^

In brief, women and men aged ≥18 years, with fluency in Swedish, a chief complaint of chest pain recorded by an ED triage nurse, a non-diagnostic first ECG and/or non-diagnostic serum markers for an acute disease requiring immediate care, and with clinical stability (Rapid Emergency Triage and Treatment System; RETTS, level orange, yellow, green, and blue)^28^ at presentation were eligible for inclusion.^25^ If inability to carry out CHT (eg, agitation, severe impaired vision, or confusion), the patient was excluded. The most common causes for being excluded were language issues (eg, could not read Swedish), tiredness, and inability to use a tablet. A detailed description why patients were not included are available in Multimedia Appendix 2 in Brandberg et al.^24^

Here, we report a pre-specified secondary analysis of the 1000 CLEOS-CPDS patients recruited (from October 1, 2017, to May 16, 2019).^25^ The study complies with the Declaration of Helsinki and was approved by the Stockholm Regional Ethical Committee (now Swedish Ethical Review Authority) (reference number 2015/1955-1). All participants provided their informed consent to participate.

Study outcomes for the current report were (1) the fraction of patients with sufficient data to calculate established risk scores to indicate the need for hospitalization or to allow for a safe disposition to home (ie, a *clinically decisive* risk score), and (2) the agreement between risk score variable data collected from CHT and from the EHR.

### Interventions

**Figure 1.**
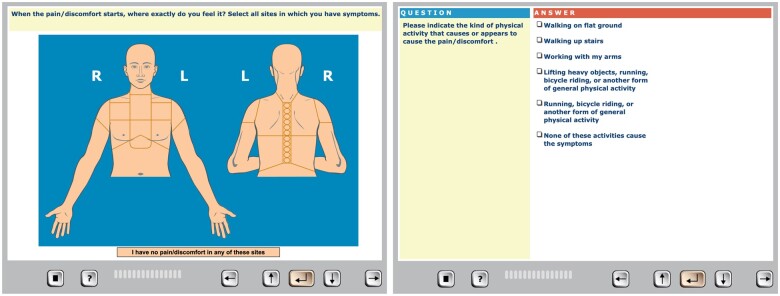
User interface and examples of questions in the CHT program on a tablet.

Medical histories were collected using the CHT software CLEOS running on tablets (iPad, Apple Inc, Cupertino, CA, United States), as described in detail and validated previously.^18^^,^^20^^,^^25^ Briefly, CLEOS is an expert system that interacts directly with the patient and employs a rule-based approach, based on consensus pathophysiology across organ systems, to facilitate the history taking process. Questions are mainly in structured text format (ie, yes/no or multiple-choice questions). Images are used for patient entries of anatomical data, eg, the site(s) of chest pain (Figure 1). A limited number of free text entries are used, when patients cannot select, for example, an appropriate answer for the quality of their pain. CLEOS tailors the interview for individual patients with a relevant line of questions that emulates an interview by a physician (examples in Supplementary Figures S1-S3). For exploratory purposes, the interview in its current form is comprehensive, and many patients will not be able to complete it during the waiting time in the ED. Since this study specifically focuses on chest pain management, the initial portion of the CHT interview included questions on chest pain characteristics, followed by questions concerning established risk factors for ACS. As previously presented, the median duration to collect HEART score was 23 (IQR 18-31) min.^24^

### Data collection

For acute chest pain management, regional guidelines recommend the use of a combination of a modified HEART score and high-sensitivity cardiac troponin assays with the 0/1h rule-out and rule-in algorithm.^29^^,^^30^ Thus, in accordance with regional guidelines,^31^ we used the traditional clinical classification of suspected anginal symptoms (ie, the 3 characteristics central chest pain, precipitated by physical or emotional exertion, and relieved by rest or nitrates)^32^ for the assessment of the History component in HEART score. Based on the number of characteristics met, the History component was categorized as highly (3 characteristics met), moderately (2 characteristics met), or slightly suspicious (none or 1 characteristic met). A *complete* risk score was defined as the availability of all medical history components of a risk score being available in the CHT or EHR datasets. We also defined a *clinically decisive* risk score in which the threshold score for “not low risk” (here to indicate need for hospitalization), ie, HEART score ≥4, EDACS ≥16, or T-MACS ≥0.05, or a rule-in troponin using the European Society of Cardiology 0/1-h algorithm,^4^ was satisfied. Details about chest pain characteristics and risk factors components for HEART score, EDACS, and T-MACS are outlined in Table 1.

**Table 1.** Variables included in risk scores, answers provided, and the agreement (inter-rater reliability) between answers when available both in the CHT database and the EHR.

	Risk score			Variable	**Answers provided (*n*)** ^a^				**Actual answers (*n*)** ^b^ **(CHT/EHR)**				Inter-rater reliability		
	HEART	EDACS	T-MACS		Neither in EHR, nor in CHT	Only in EHR, not in CHT	Only in CHT, not in EHR	In both EHR and CHT	Y/Y	N/N	Y/N	N/Y	Kappa (95% CI)	Strength Landis and Koch	Strength McHugh
**Chest pain characteristics**	X			Central chest pain	0	2	38	960	242	317	363	38	0.25 (0.20-0.29)	Fair	Minimal
	X			Provoked by physical exertion	37	36	404	523	61	326	42	94	0.31 (0.22-0.40)	Fair	Minimal
	X			Provoked by emotional stress	74	3	896	27	10	4	0	13	0.19 (0.00-0.37)	Slight	None
	X			Relieved by rest	78	4	852	66	23	16	2	25	0.26 (0.09-0.43)	Fair	Minimal
	X			Relieved by nitrates	76	11	810	103	40	34	11	18	0.44 (0.27-0.61)	Moderate	Weak
		X	X	Diaphoresis	78	128	276	518	35	369	96	18	0.28 (0.18-0.37)	Fair	Minimal
		X		Pain radiating to arm	40	19	606	335	120	165	12	38	0.70 (0.62-0.78)	Substantial	Moderate
			X	Pain radiating to right arm	40	19	606	335	23	286	15	11	0.60 (0.46-0.74)	Moderate	Moderate
		X		Pain radiating to shoulder	48	11	740	201	32	133	10	26	0.53 (0.39-0.66)	Moderate	Weak
			X	Pain radiating to right shoulder	48	11	740	201	3	179	10	9	0.19 (−0.04 to 0.42)	Slight	None
		X		Pain worsened with inspiration	90	37	548	325	109	152	17	47	0.60 (0.52-0.69)	Moderate	Moderate
		X		Pain is reproduced by palpation	92	48	595	265	55	134	13	63	0.39 (0.29-0.50)	Fair	Minimal
			X	Worsening or crescendo angina	137	97	557	209	32	135	24	18	0.47 (0.33-0.61)	Moderate	Weak
			X	Pain associated with vomiting	179	33	688	100	9	69	21	1	0.35 (0.16-0.54)	Fair	Minimal
**Risk factors**	X			Atherosclerotic disease	67	82	443	408	111	276	8	13	0.88 (0.83-0.93)	Almost perfect	Strong
		X		Known CAD	79	70	466	385	107	263	6	9	0.91 (0.86-0.95)	Almost perfect	Almost perfect
	X	X		Diabetes mellitus	128	84	495	293	51	228	3	11	0.85 (0.77-0.93)	Almost perfect	Strong
	X	X		Current smoker	29	181	85	705	52	618	2	33	0.72 (0.64-0.81)	Substantial	Moderate
	X	X		Family history of premature CAD	109	117	310	464	72	319	55	18	0.57 (0.48-0.65)	Moderate	Weak
	X	X		Hypertension	78	157	310	455	208	187	30	30	0.74 (0.67-0.80)	Substantial	Moderate
	X	X		Hypercholesterolemia	200	160	361	279	73	189	4	13	0.85 (0.79-0.92)	Almost perfect	Strong
	X			Obesity	0	0	924	76	34	25	7	10	0.55 (0.36-0.74)	Moderate	Weak

Agreement between CHT and EHR is presented as inter-rater reliability calculated in cases where answers were available in both CHT and EHR. Obesity: BMI >30 kg/m^2^ based on self-reported weight and height. Strength presented according to Landis and Koch^33^ and McHugh.^34^

Abbreviations: CAD = coronary artery disease; CHT = computerized history taking; CI = confidence interval; EHR = electronic health record.

a This represents any number of answers in CHT and EHR.

b This represents the number of actual answers (ie, yes or no; reported as Y or N) provided by CHT and EHR.

In the ED cardiology unit or the inpatient day-care unit, a research assistant invited patients to participate in the study. To ensure consistency in the inclusion process, all research staff were trained using a standardized protocol including specific information to be communicated to patients. The inclusion process was regularly monitored by a senior member of the research team, who also resolved any uncertainties to ensure consistency. After informed consent had been obtained, participants were provided with a tablet equipped with the CHT program. To ensure that the patient could manage the CHT program, a research assistant remained at the side of the patient while the first questions regarding demography were asked and answered. Patients unable to answer these questions were not included in the study. History taking by CHT did not interfere with standard management in any way. CHT was performed during waiting times and could take place both before, and after seeing a physician. The CHT could be interrupted, eg, due to a radiographic examination or blood sampling, and resumed afterwards. The level of privacy and ambient noise during the interview varied as patients stayed in the ED waiting room, or ED spaces separated by curtains or solid walls. The ED staff did not at any time have access to any information collected with CHT. The CHT was discontinued either when it was fully completed, the patient chose to stop for any reason, or when the patient was discharged to home or admitted to a ward or day-care unit. Reasons for discontinuation of CHT were recorded by research staff.

Relevant data for the present work were collected from the EHR (Take Care, CompuGroup Medical Sweden AB, Solna, Sweden). This EHR system features a semi-structured patient journal format with predefined fields and checkboxes, as well as free-text fields. However, the EHR system did not provide any structured way for detailed reporting of the risk scores. Variables to be collected from the CHT database and the EHR were predefined, including their interpretation, to enable a conversion into a binary format (yes/no answers) corresponding to variables in the 3 risk scores used in this study. Data extraction was performed manually by research staff, including medical students and research nurses, according to a specific protocol. Any inconsistencies were discussed and resolved with a senior cardiologist. Further information of the general ED population (age and sex) was collected from the EHR system, using QlikView version 12.10 (QlikTech International AB, Lund, Sweden) by H.B.

### Statistical analyses

The study sample size was determined by the planned diagnostic study within CLEOS-CPDS, where 1000 participants are required to achieve the targeted precision of sensitivity and specificity of ±0.03 (3%) for an ACS.^25^

**Figure 2.**
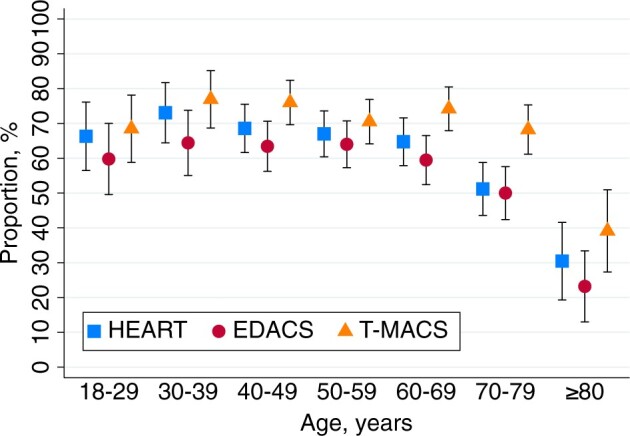
Proportions of calculations of a complete risk score using CHT data, stratified by age. Data are presented as mean values with 95% CIs.

Data are presented as mean values ± SD or 95% confidence intervals, median values and IQR, or proportions, as appropriate. The population was stratified into 7 age groups (Figure 2) when comparing proportions from whom a *complete* risk score could be calculated, and into 3 age groups (18-49, 50-69, and ≥70 years). Also, stratification was done based on the time from arrival at the ED to CHT start (0-59, 60-119, 120-179, and ≥180 min). Age groups and groups based on time of arrival were derived after data collection to provide enough numbers to assess the agreement between CHT and EHR data. For group comparisons, the Pearson’s Chi-squared test and the Kruskal-Wallis test followed by Dunn’s pairwise comparison and Bonferroni adjustments were used. Cohen’s kappa statistics was used to assess the agreement between CHT and physician-acquired history taking as a measure of inter-rater reliability. This involved determining the degree to which these 2 methods assigned the same score value to the same variable for an individual patient.^34^ Given that inter-rater reliability is frequently applied in analysis of self-reported data,^35–38^ employing Cohen’s kappa also allowed our study to align with and be compared to previous research. It is important to note that inter-rater reliability calculations were feasible only in cases where a specific observation was recorded by both CHT and the EHR for the same patient. We interpreted kappa values according to Landis and Koch^33^ and McHugh.^34^ To account for the possibility that a positive finding not documented in the EHR does not automatically indicate that physicians overlooked asking about or including this information in their decision making, we conducted a sensitivity analysis for inter-rater reliability. In this analysis, we treated missing data, such as unmentioned instances of diaphoresis, as if these conditions were confirmed to be negated. Analyses were conducted using STATA software, version 14.2 (StataCorp, College Station, TX, United States).

## Results

### General

During the study period, 13 044 patients presented to the ED at Danderyd University Hospital, representing the general chest pain population. Of these, a total of 1000 patients were enrolled (convenience sample), during time periods with on duty research staff (office hours, evenings, and weekends). Baseline characteristics collected by CHT, and selected ED data are presented in Table 2.

**Table 2.** Baseline characteristics for the study population.

Characteristic	Value	*n*
Age, years	55.3 ± 17.4	1000
Sex (females)	456 (46%)	1000
Body mass index, kg/m^2^	26.4 ± 4.7	1000
Diabetes mellitus type 1 or 2	62 (8%)	788
Ongoing lipid lowering medication	128 (20%)	640
Hypertension	316 (41%)	765
Family history of coronary artery disease	200 (26%)	774
Known coronary artery disease	133 (16%)	851
History of angina pectoris	88 (10%)	850
History of myocardial infarction	82 (10%)	850
History of percutaneous coronary intervention	76 (9%)	850
History of coronary artery bypass graft	18 (2%)	833
Current smoker	59 (7%)	790
Region of birth		1000
Nordic countries	829 (83%)	
Europe (outside the Nordic countries)	46 (5%)	
Outside Europe	125 (13%)	
Occupational status		1000
Active worker (employed, student)	616 (62%)	
Not at work (unemployed, on sick leave)	69 (7%)	
Retired	315 (32%)	
Arrived at ED by ambulance	189 (21%)	917
Ongoing chest pain during CHT	544 (61%)	892
Time from ED arrival to interview starts, min		1000
0-59	136 (14%)	
60-119	398 (40%)	
120-179	252 (25%)	
≥180	214 (21%)	
Admitted to the ward or day-care unit	528 (53%)	999
Ward	203 (20%)	
Cardiology unit	188 (19%)	
Non-cardiology unit	15 (2%)	
Cardiology day-care-unit	325 (33%)	
Day-care unit then to ward	33 (3%)	
Day-care unit then sent home	292 (29%)	

Data are presented as mean values ±SD or *n* (%), as appropriate. Data derived from CHT except “Time from ED arrival to interview start” and “Admitted to the ward or day-care unit,” which were derived from the EHR. Body mass index is based on self-reported height and weight. The *n* varies as all participants did not reach this question during the CHT interview. See text for details.

Abbreviations: CHT = computerized history taking; ED = emergency department.

Of note, age, and sex distribution of the study population (mean age 55.3 ± 17.4 years; 46% women) were comparable to the general chest pain population at Danderyd University Hospital (mean age 57.6 ± 19.1 years; 49% women). In the study population, 20% of the patients arrived at the ED by ambulance, compared to 30% in the general chest pain population (with the same RETTS level as in the study population). Furthermore, 53% of the study population were admitted to a ward or day-care unit, as compared 46% in the general chest pain population. In the study population, almost two thirds reported ongoing chest pain during the CHT interview. Median length of stay in the ED was 250 (IQR 216-283) min during the study period, and 53% of the patients started the interview within 120 min after ED arrival.

### Calculation of risk scores from CHT and EHR data

*Clinically decisive* risk scores by HEART score, EDACS, and T-MACS could be calculated respectively in 75%, 74%, and 83% from CHT data. The same scores could be calculated for respectively 31%, 7%, and 25% from EHR data (Table 3). The fractions of the study population for whom a *complete* risk score could be calculated, and details of available medical history components (chest pain characteristics and risk factors) are presented in Table 3.

**Table 3.** The proportion of 1000 participants for whom a clinically decisive risk score and a complete risk score (and its individual components) could be calculated from CHT and EHR data, respectively.

	**HEART**, n (%)	**EDACS**, n (%)	**T-MACS**, *n* (%)	**Any of HEART, EDACS, or T-MACS**, *n* (%)
Clinically decisive risk score				
CHT	751 (75)	735 (74)	834 (83)	900 (90)
EHR	308 (31)	103 (10)	253 (25)	420 (42)
Complete risk score				
CHT	669 (67)	612 (61)	701 (70)	802 (80)
EHR	0 (0)	12 (1)	14 (1)	26 (3)
Chest pain characteristics components				
CHT	905 (91)	778 (78)	701 (70)	–
EHR	0 (0)	29 (3)	14 (1)	–
Risk factors components				
CHT	675 (68)	675 (68)	N/A	–
EHR	375 (38)	351 (35)	N/A	–

Values represent numbers (%). A *complete* risk score was considered if all components of a risk score were available. A *clinically decisive* risk score also included cases where the accumulated score value reached the threshold for “not low risk” (to indicate the need for hospitalization, ie, HEART score ≥4, EDACS ≥16, or T-MACS ≥0.05) or a rule-in troponin using the European Society of Cardiology 0/1-hour algorithm.^4^ See text for further details. Chest pain characteristics and risk factors components are outlined in Table 1.

Abbreviations: CHT = computerized history taking; EHR = electronic health record; HEART = History, ECG, Age, Risk factors and Troponin; EDACS = Emergency Department Assessment of Chest Pain; T-MACS = Troponin-only Manchester acute coronary syndromes; N/A = not applicable.

**Figure 3.**
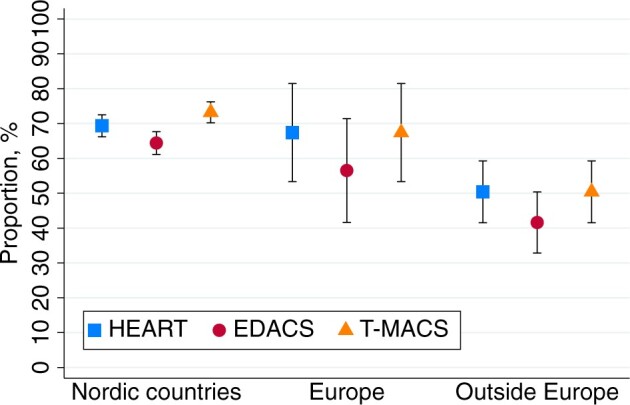
Proportions of calculations of a complete risk score using CHT data, stratified by birth region. Data are presented as mean values with 95% CIs. Europe: birth region in Europe outside the Nordic countries.

**Figure 4.**
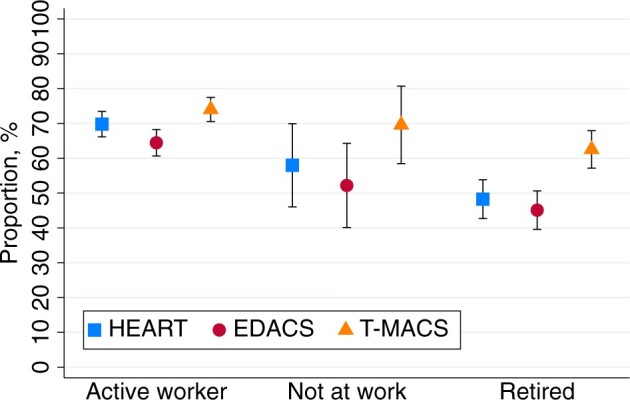
Proportions of calculations of a complete risk score using CHT data, stratified by occupation status. Data are presented as mean values with 95% CIs.

A *complete* HEART score and a *complete* EDACS could be calculated more often in males than in females (71% vs 62%, *P* = .002, and 65% vs 57%, *P* = .006, respectively). No sex difference was found for calculating a *complete* T-MACS (71% vs 70%, *P* = .713). The proportion of patients for whom *complete* risk scores could be calculated was lower for patients 80 years or older (however, less pronounced for T-MACS), as compared to younger patients (Figure 2), was lower for birth region outside Europe, as compared to birth region in Nordic countries (Figure 3), and was higher for active workers, as compared to retired persons (Figure 4). Risk score calculation for patients arriving by ambulance, as compared with walk-in patients was possible less often for *complete* HEART score (67% vs 74%, *P* = .005), *complete* EDACS (58% vs 69%, *P* = .001), and *complete* T-MACS (68% vs 78%, *P* = .003). This lower completion rate was associated with a higher incidence of tiredness as the reason for discontinuing the CHT interview among patients arriving by ambulance, as compared to walk-in patients (28% vs 19%, *P* = .006).

Time from arrival to start of CHT did not affect the availability of data for calculating a *complete* score, except for a *complete* T-MACS score. Patients starting CHT within 60 min of arrival (*n* = 136) *completed* a T-MACS score more frequently than patients starting CHT ≥180 min (*n* = 214) after arrival (77% vs 64%, *P* = .020). No differences in the fraction of patients with *complete* scores were found for patients with and without ongoing chest pain during the CHT in the ED, or between patients discharged to home or admitted to a day-care unit or ward (Supplementary Table S1).

In patients for whom *complete* risk scores by HEART score, EDACS, or T-MACS could not be calculated from CHT data, the main reasons for discontinuing the interview were that the patient felt tired (26%-29%) or was discharged from the ED (24%-27%). The main reasons for discontinuing the interview prior to completion, but *with* sufficient data to calculate a full risk score were the same in cases where a risk score could not be calculated. However, feeling tired or difficulty using a tablet were more frequently reported reason for discontinuation in the group without sufficient data for risk score calculation. All reasons for discontinuing CHT are presented in Table 4.

**Table 4.** Proportion of patients where a complete risk score could be calculated, and reasons for discontinuing the CHT.

	**All**, n (%)	**Complete HEART**, *n* (%)		*P*	**Complete EDACS**, *n* (%)		*P*	**Complete T-MACS**, *n* (%)		*P*
**Reasons**		No	Yes		No	Yes		No	Yes	
Completed the full CHT interview	209 (21)	4 (1)	205 (31)	<.001	14 (4)	195 (32)	<.001	14 (5)	195 (28)	<.001
Discharge to home	234 (23)	81 (25)	153 (23)	.574	99 (26)	135 (22)	.208	80 (27)	154 (22)	.102
Getting tired	204 (20)	109 (29)	95 (16)	<.001	107 (28)	97 (16)	<.001	78 (26)	126 (18)	.004
Reason for discontinuation not stated	143 (14)	51 (15)	92 (14)	.481	56 (14)	87 (14)	.924	37 (12)	106 (15)	.256
End of research staff work shift	51 (5)	24 (7)	27 (4)	.030	29 (7)	22 (4)	.007	18 (6)	33 (5)	.388
Admission/transfer within the hospital	41 (4)	21 (6)	20 (3)	.001	24 (6)	17 (3)	.008	19 (6)	22 (3)	.019
Difficulty to use tablet	30 (3)	25 (8)	5 (1)	<.001	25 (6)	5 (1)	<.001	27 (9)	3 (0)	<.001
Physical examination or investigational procedure	26 (3)	8 (2)	18 (3)	.798	9 (2)	17 (3)	.657	6 (2)	20 (3)	.441
Technical issues	25 (3)	4 (1)	21 (3)	.066	4 (1)	21 (3)	.018	5 (2)	20 (3)	.274
Not relevant/too many questions	15 (2)	5 (2)	10 (1)	.985	6 (2)	9 (1)	.923	4 (1)	11 (2)	.783
Acute medical condition/measure	14 (1)	8 (2)	6 (1)	.054	9 (2)	5 (1)	.097	6 (2)	8 (1)	.286
Other	8 (1)	5 (2)	3 (0)	.076	6 (2)	2 (0)	.035	5 (2)	3 (0)	.043
**Total**	1000 (100)	331 (33)	669 (67)		388 (39)	612 (61)		299 (30)	701 (70)	

Values represent numbers and column percentages for all 1000 participants. P values represent differences within rows (No/Yes). Reasons for discontinuation prior to completion of a full interview are available in Supplementary Table S2. For a more detailed description of reasons for discontinuations, see Brandberg et al.^24^

Abbreviations: CHT = computerized history taking; EDACS = Emergency Department Assessment of Chest Pain; HEART = History, ECG, age, risk factors, troponin; T-MACS = Troponin-only Manchester acute coronary syndromes.

### Completion of a full interview

Most patients (*n* = 791) did not complete the full CHT interview (including all organ systems).^25^ However, this did not affect the possibility of achieving a *complete* risk score as the questions required for this were asked early in the interview. The main reasons for not completing a full interview were discharge from the ED (*n* = 234, 30%) and fatigue (*n* = 204, 26%) (Supplementary Table S2). Differences for discontinuing prior to a full interview in patients aged 18-69 years (*n* = 573) vs ≥70 years (*n* = 218) are presented in Supplementary Table S2. No difference by sex was found. Patients born outside Europe more often discontinued due to tiredness as compared with patients born in the Nordic countries or Europe (30% vs 19/13%, *P* = .013 and .026, respectively).

### Agreement between CHT and EHR data for specific components in risk scores

Data for observations in the CHT database and the EHR and their agreement are presented in Table 1. The agreement between CHT and EHR data (reported as inter-rater reliability) for variables included in chest pain characteristics was slight to moderate (kappa 0.19-0.70) and for risk factor variables moderate to almost perfect (kappa 0.55-0.91). There were no differences in inter-rater reliability by sex or birth region. There were differences between CHT and EHR data for reported pain radiating to the right shoulder (age group 50-69 vs ≥70 years) and for reported diabetes mellitus (age groups 18-49 and 50-69 vs ≥70 years). Sensitivity analyses performed to account for missing data supported our findings on the agreement between CHT and EHR data (Supplementary Table S3).

### Potential impact on clinical management

**Figure 5.**
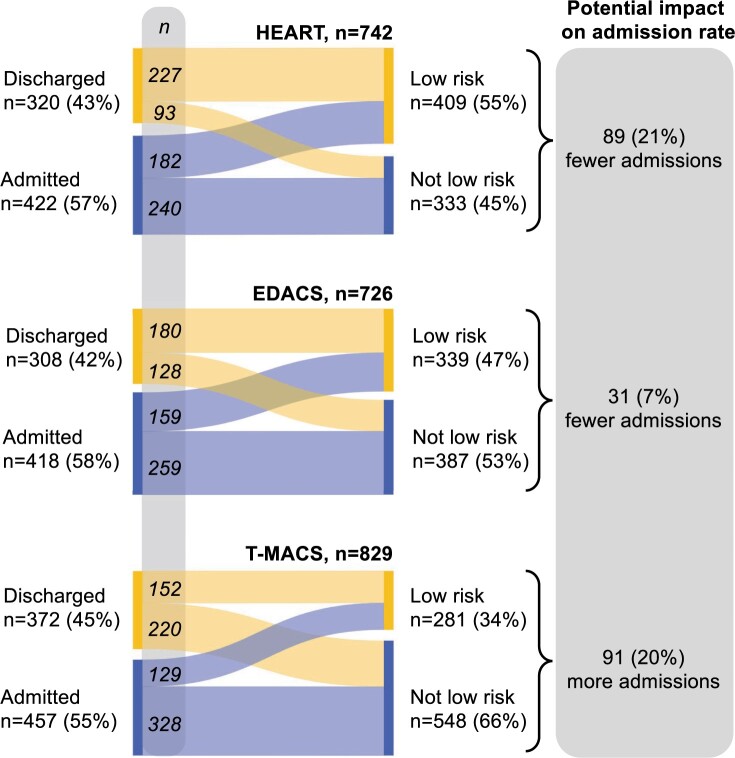
Reclassifications if risk scores derived from CHT had been used for management instead of standard care, and the potential impact on admission rates. Standard care (left) vs risk scores using CHT (right). Analysis made in cases with sufficient data to calculate a *clinically decisive* risk score and available data on disposition in the ED. Flows indicate patient transitions from discharged or admitted to low risk and not low risk categories, had risk scores derived from CHT been used. Admission was defined as admission to a ward or cardiology inpatient day-care-unit and discharged as sent home from the ED.

Reclassifications if risk scores derived from CHT had been used instead of standard care for patient management, and the potential impact on admission rates are presented in Figure 5. There were no fatal events within 7 days among patients reclassified from admission to a low-risk category.

## Discussion

Only a few studies have reported on the use of CHT in an acute setting.^19^^,^^21^^,^^22^^,^^24^ The data presented here show that CHT can collect data directly from patients to calculate risk scores by the HEART score, EDACS, and T-MACS for the majority of an ED population with acute chest pain. These risk score can be calculated from a far greater fraction by using CHT (74%-83%) than from EHR data (10%-31%). Thus, the use of CHT may provide a more complete medical history, facilitating risk stratification of patients with acute chest pain.

The patient-by-patient agreement (inter-rater reliability) of information obtained from CHT and data entered in EHR by physician-acquired history taking was high for traditional risk factors, especially for those with distinct diagnostic criteria (eg, diabetes mellitus or hypertension), and low to moderate for more subjective information such as chest pain characteristics (eg, provoking factors or pain radiating). This study may be the first to assess inter-rater reliability between medical history data collected by CHT and by physician-acquired history taking recorded in EHR. Our findings are in line with inter-rater reliability studies for self-reported questionnaires. Thus, a cohort study found high agreement between self-reports and physician reports for hypertension, diabetes mellitus, hyperlipidemia, and hyperuricemia.^36^ Patients are reliable reporters of a previous diagnosis of angina pectoris, myocardial infarction, and diabetes mellitus.^37^^,^^38^ This suggests that traditional risk factor data collected with CHT is comparable to EHR data, while CHT data provide more information for chest pain characteristics than EHR data^19^ contributing to the observed low to moderate agreement.

Our findings may reflect insufficient documentation in the EHR of clinical conditions.^19^^,^^39^ However, relevant conditions and symptoms may be observed and considered in the evaluation of the patient, without being recorded in the EHR, as may be suggested by our findings (Supplementary Table S3). This notwithstanding, the proportion of patient files with the relevant variables recorded was considerably higher in CHT records, as compared to EHR. This indicates that CHT can provide more information on potentially important issues. Whether and the extent to which these findings may affect management and outcome remain to be assessed in clinical outcome studies.

In this study, discharge from the ED to home and fatigue were the most common causes for discontinuing CHT prior to completing data elements needed for risk score calculation. We observed some differences in completion of risk scores based on age, sex, birth region, and occupational status, which extend our previous findings.^24^ It is possible that the proportion of patients with sufficient data for calculating a risk score could be enhanced by improved design of the CHT user interface, by removing questions that are not clinically revealing, and by shortening delays between arrival in the ED and the start of CHT. However, apart from a small difference in completion of a T-MACS score between early (<60 min) and late (≥180 min) CHT start after ED arrival, no difference was found for completion of the scores by time from arrival to CHT start. Furthermore, we observed that CHT score completion was better for T-MACS than for HEART and EDACS among older patients. This finding is likely because T-MACS is calculated from chest pain characteristics and biomarkers (ECG, blood pressure, and troponin), without incorporating medical history. As described in the Methods section, the CHT interview begins with questions exploring chest pain characteristics before shifting to medical history and other known risk factors for ACS, explaining this disparity. Despite limitations, CHT as compared with physician-acquired history taking appears to offer advantages for collecting sufficient data for risk stratification in the acute setting. Long-term follow-up and further analyses of the current study may reveal which questions are most critical to ask, thereby potentially shortening the CHT interview process.

The use of CHT can significantly impact the management of patients in the ED, as illustrated in Figure 5. Our results indicate that the HEART score and EDACS populated with CHT data, reclassify patients to a considerable extent (7%-21%) between admission (ie, rule-in) and the low-risk category (ie, rule-out), suggesting that more efficient management is possible to achieve. Conversely, the use of T-MACS with CHT data may lead to increased number of admissions (20%), suggesting that T-MACS may be less specific than the other 2 risk scores. Overall, there is potential for this approach to enhance management effectiveness and resource utilization for the substantial portion of ED patients with chest pain. However, conclusions on this issue await future analyses with long-term outcome data, which are subject of ongoing studies.

The strengths of this study are a large study population representative of an ED chest pain population, and a prospective cohort design including 3 established validated risk score algorithms. Also, the generic layout of this CHT program may allow the results to be extrapolated to other clinical conditions and settings. However, several limitations warrant discussion. First, patients not able to perform CHT or with limited Swedish language proficiency were not included, which may affect generalizability. However, CHT can be available in any language, which would eliminate these limitations. Additionally, the tool could serve as a component in promoting equitable healthcare for individuals with limited ability to speak the local language, a challenge that has been previously highlighted.^40^^,^^41^ Second, a possible selection bias might have been introduced as patients had different socioeconomic background, level of language skills, or experience of using a tablet. Patients born outside Europe more often aborted the CHT due to tiredness, which could be due to being less used to Swedish language or possibly having a lower level of education, making the interview more demanding. These issues were addressed in a previous utility study,^24^ and warrant development of more user-friendly CHT software for future clinical implementation. Furthermore, despite our efforts to standardize the inclusion procedure, additional selection bias may have been introduced as multiple research assistants participated in patient recruitment, and a degree of subjective judgment was permitted to determine a patient’s ability to carry out CHT. Third, regional guidelines recommend using the HEART score in managing chest pain patients in the ED. However, since it is not required to provide a structured documentation of risk scores in the EHR, it remains possible that a more systematic approach for collection of risk scores in the EHR might have yielded different results. This highlights an area for further research into the effects of structured data collection on clinical management. Fourth, we did not double-check information extracted from the EHR; however, this extraction followed a predefined protocol, with senior research members available to address any inconsistencies. Finally, further development of the CHT software to include only pivotal questions could further enhance the efficiency of medical history taking, while a broadened clinical focus may provide the diagnostic capability for a range of acute conditions, eg, aortic dissection and pulmonary embolism.

## Conclusion

CHT can acquire and document data for risk stratification of chest pain using established risk scores (HEART score, EDACS, and T-MACS) in most patients attending the ED and achieve this goal in a substantially larger number of patients, as compared with EHR data. Furthermore, the agreement between CHT and physician-acquired history taking is high for traditional risk factor variables and low to moderate for pain characteristics. These findings suggest that CHT is a promising technology for facilitating risk stratification and safer management of ED patients with acute chest pain.

## Supplementary Material

ocae110_Supplementary_Data

## Data Availability

The data underlying this article will be shared on reasonable request to the corresponding author, H.B.
