# Correction to: Are medical history data fit for risk stratification of patients with chest pain in emergency care? Comparing data collected from patients using computerized history taking with data documented by physicians in the electronic health record in the CLEOS-CPDS prospective cohort study

**DOI:** 10.1093/jamia/ocae252

**Published:** 2024-09-28

Helge Brandberg, Carl Johan Sundberg, Jonas Spaak, Sabine Koch, Thomas Kahan, Are medical history data fit for risk stratification of patients with chest pain in emergency care? Comparing data collected from patients using computerized history taking with data documented by physicians in the electronic health record in the CLEOS-CPDS prospective cohort study, *Journal of the American Medical Informatics Association*, Volume 31, Issue 7, July 2024, Pages 1529–1539, https://doi.org/10.1093/jamia/ocae110

In the originally published manuscript, the authors found errors in the reporting of sex distribution. The distribution of sex was incorrectly reported as n = 544 (54%) female. The correct figure is n = 456 (46%) female. This also affected the reporting of one of the risk scores (EDACS). However, the authors would like to emphasize that these emendations corrections have a minor effect on the results, but do not affect the paper’s major findings or conclusions in any significant way.

The following changes have been made to:

Page 1, Abstract, Results: “54% women” should be “46% women”.

Page 1, Abstract, Results: “HEART score, EDACS, and T-MACS could be calculated in 75%, 74%, and 83% by CHT and in 31%, **7%**, and 25% by EHR, respectively.” should be HEART score, EDACS, and T-MACS could be calculated in 75%, 74%, and 83% by CHT and in 31%, **10%**, and 25% by EHR, respectively.

Page 2, Graphical abstract: “54% women” should be “46% women”.

Page 2, Graphical abstract, Possible risk score calculation: “7–31%”, should be “10–31%”.

Page 4, Results, General, 2nd para: “54% women”, should be “46% women”.

Page 6, Table 2: Value for Sex (females) “544 (54%)”, should be “456 (46%)”.

Page 6, Results, Calculations of risk scores from CHT and EHR data, 2nd para: The scentence “A complete HEART score and a complete EDACS could be calculated more often in **females** than in **males**” should be corrected to “A complete HEART score and a complete EDACS could be calculated more often in **males** than in **females**”.

Page 7, Table 3, Reports for a Clinically decisive risk score from CHT for EDACS: “744 (74)”, should be “735 (74)”.

Page 7, Table 3, Reports for a Clinically decisive risk score from EHR for EDACS: “66 (7)”, should be “103 (10)”.

Page 9, Figure 5: Figures for EDACS have been corrected.

Figure 5 should read:

**Figure 5.**
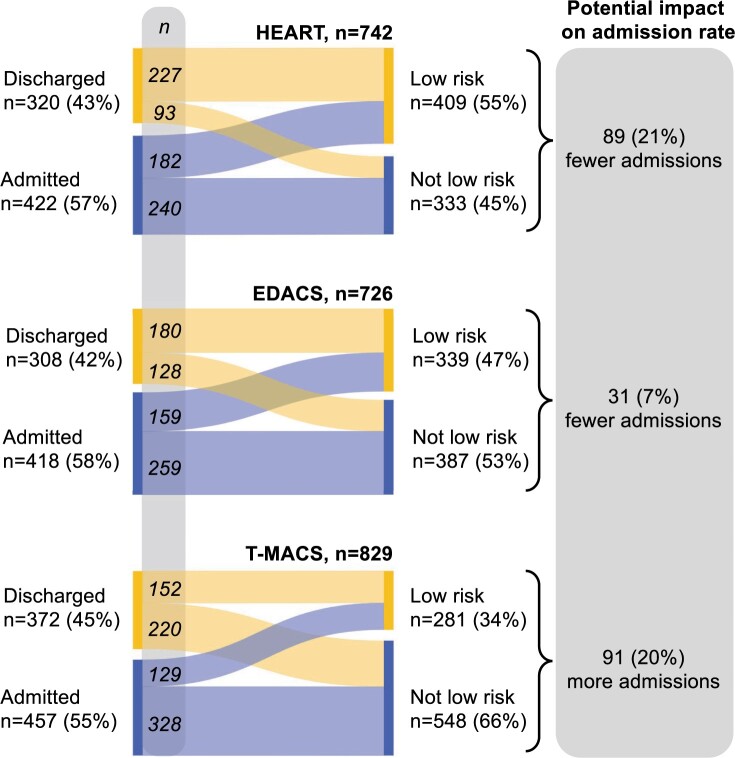
Reclassifications if risk scores derived from CHT had been used for management instead of standard care, and the potential impact on admission rates. Standard care (left) vs risk scores using CHT (right). Analysis made in cases with sufficient data to calculate a *clinically decisive* risk score and available data on disposition in the ED. Flows indicate patient transitions from discharged or admitted to low risk and not low risk categories, had risk scores derived from CHT been used. Admission was defined as admission to a ward or cardiology inpatient day-care-unit and discharged as sent home from the ED.

**Figure 5.**
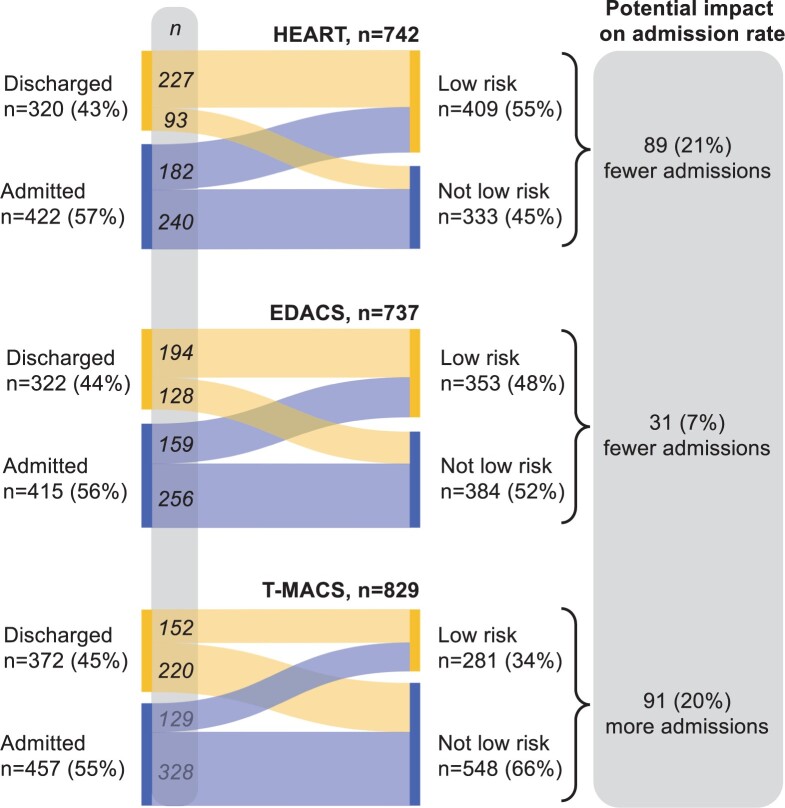
Reclassifications if risk scores derived from CHT had been used for management instead of standard care, and the potential impact on admission rates. Standard care (left) vs risk scores using CHT (right). Analysis made in cases with sufficient data to calculate a *clinically decisive* risk score and available data on disposition in the ED. Flows indicate patient transitions from discharged or admitted to low risk and not low risk categories, had risk scores derived from CHT been used. Admission was defined as admission to a ward or cardiology inpatient day-care-unit and discharged as sent home from the ED.

Instead of:

Please note that these corrections do not alter the potential impact on admission rates.

In the Supplementary data, Supplementary Table 1, 2nd line: “Male”, should be “Female”; 3rd line: “Female”, should be “Male”.

