# Supplementary material for: Are medical history data fit for risk stratification of patients with chest pain in emergency care? Comparing data collected from patients using computerized history taking with data documented by physicians in the electronic health record in the CLEOS-CPDS prospective cohort study
Source: J Am Med Inform Assoc. 2024 May 23;31(7):1529–39. doi: 10.1093/jamia/ocae110 (PMC11187423; doi:10.1093/jamia/ocae110)
Supplement: ocae110_Supplementary_Data [file ocae110_supplementary_data.pdf]

Are medical history data fit for risk stratification of patients with chest pain in emergency care? Comparing data collected from patients using computerized history taking with data documented by physicians in the electronic health record in the CLEOS-CPDS prospective cohort study

Supplementary File

Supplementary Tables

Supplementary Table 1. Group comparisons of possible external influence to calculate a complete risk score 2

Supplementary Table 2. Reasons for discontinuing the interview stratified by age in patients not completing the full interview 3

Supplementary Table 3. Sensitivity analysis of the agreement (inter-rater reliability) between answers in the CHT and the EHR 4

Supplementary Figures

Supplementary Figure 1. Overview of questions in the computerised history-taking interview relevant for chest pain relief by rest 5

Supplementary Figure 2. Overview of questions in the computerised history-taking interview relevant for chest pain relief by nitrates 7

Supplementary Figure 3. Overview of questions in the computerised history-taking interview relevant for worsening/crescendo angina pectoris 9

Supplementary Table 1. Group comparisons of possible external influence to calculate a complete risk score.

**Characteristic**	**All**<br>n	**HEART**<br>n (%)	**EDACS**<br>n (%)	**T-MACS**<br>n (%)
Male	544	387 (71)	354 (65)	384 (71)
Female	456	282 (62)	258 (57)	317 (70)
**P**		.002	.006	.713
Arrival by ambulance	189	127 (67)	110 (58)	129 (68)
Not arrival by ambulance	728	541 (74)	501 (69)	571 (78)
**P**		.050	.006	.003
Time to interview start (min)	1000			
0-59	136	96 (71)	89 (65)	105 (77)
60-119	398	272 (68)	248 (62)	280 (70)
120-179	252	166 (66)	151 (60)	180 (71)
≥180	214	135 (63)	124 (58)	136 (64)
**P**		.438–1.000	.483–1.000	.020–1.000*
No ongoing chest pain	348	256 (74)	243 (70)	277 (80)
Ongoing chest pain	544	412 (76)	368 (68)	423 (78)
**P**		.466	.494	.514
Admitted to ward or day-care unit	528	363 (69)	332 (63)	366 (69)
Not admitted	471	306 (65)	280 (59)	335 (71)
**P**		.205	.267	.533
**All**	1000	669 (67)	612 (61)	701 (70)

Values represent numbers (%). Missing values for type of arrival (n=83), self-reported ongoing chest pain (n=108) and admission (n=1). * Denotes a significant difference between patients with time from ED arrival to interview start between 0-59 mins and >180 min (P=.020). HEART: History, ECG, age, risk factors, troponin. CHT: Computerised history taking. EDACS: Emergency department assessment of chest pain. T-MACS: Troponin-only Manchester acute coronary syndromes.

Supplementary Table 2. Reasons for discontinuing the interview stratified by age in patients not completing the full interview.

**Reasons**	**All** n (%)	**18-69 years** n (%)	**≥70 years** n (%)	**P**
Discharge from ED	234 (30)	198 (35)	36 (17)	<.001
Gets tired	204 (26)	144 (25)	60 (28)	.492
Missing/not stated	143 (18)	111 (19)	32 (15)	.125
End of research staff work shift	51 (6)	38 (7)	13 (6)	.732
Admission/transfer	41 (5)	20 (3)	21 (10)	<.001
Difficulty to use tablet	30 (4)	5 (1)	25 (11)	<.001
Examination	26 (3)	19 (3)	7 (3)	.941
Technical issues	25 (3)	17 (3)	8 (4)	.614
Not relevant/to many questions	15 (2)	8 (1)	7 (3)	.095
Acute medical condition/measure	14 (2)	7 (1)	7 (3)	.058
Other	8 (1)	6 (1)	2 (1)	.871
**Total**	791 (100)	573 (72)	218 (28)	

Values represent numbers (%) for 791 patients. ED: emergency department

Supplementary Table 3. Sensitivity analysis of the agreement (inter-rater reliability) between answers in the CHT and the EHR.

Variable	All patients		Patients with a missing value in the EHR replaced	
	n	Kappa (95% CI)	n	Kappa (95% CI)
**Chest pain characteristics**				
Central chest pain	960	0.25 (0.20–0.29)	998	0.23 (0.19–0.27)
Provoked by physical exertion	523	0.31 (0.22–0.40)	927	0.28 (0.21–0.36)
Provoked by emotional stress	27	0.19 (0.00–0.37)	923	0.27 (0.13–0.41)
Relieved by rest	66	0.26 (0.09–0.43)	918	0.13 (0.07–0.20)
Relieved by nitrates	103	0.44 (0.27–0.61)	913	0.56 (0.45–0.66)
Diaphoresis	518	0.28 (0.18–0.37)	794	0.21 (0.14–0.28)
Radiation to arm	318	0.70 (0.62–0.78)	941	0.55 (0.49–0.62)*
Radiation to right arm	318	0.60 (0.46–0.74)	941	0.42 (0.30–0.54)
Radiation to shoulder	201	0.53 (0.39–0.66)	941	0.19 (0.12–0.26)*
Radiation to right shoulder	201	0.19 (-0.04–0.42)	941	0.08 (-0.02–0.18)
Pain occurred or worsened with inspiration	325	0.60 (0.52–0.69)	873	0.47 (0.40–0.54)
Pain is reproduced by palpation	265	0.39 (0.29–0.50)	860	0.33 (0.24–0.41)
Worsening or crescendo angina	175	0.47 (0.33–0.61)	766	0.23 (0.15–0.31)*
Pain associated with vomiting	265	0.35 (0.16–0.54)	788	0.13 (0.05–0.20)
**Risk factors**				
Atherosclerotic disease	408	0.88 (0.83–0.93)	851	0.82 (0.77–0.88)
Known CAD	385	0.91 (0.86–0.95)	851	0.84 (0.78–0.89)
Diabetes mellitus	293	0.85 (0.77–0.93)	788	0.81 (0.73–0.89)
Current smoker	675	0.72 (0.64–0.81)	790	0.70 (0.61–0.78)
Family history of premature CAD	464	0.57 (0.48–0.65)	774	0.40 (0.33–0.48)*
Hypertension	455	0.74 (0.67–0.80)	765	0.61 (0.56–0.67)*
Hypercholesterolaemia	279	0.85 (0.79–0.92)	640	0.62 (0.54–0.70)*
Reported obesity	76	0.55 (0.36–0.74)	1000	0.24 (0.17–0.32)*

Assessment of the agreement between CHT and EHR when a missing statement in EHR was classified as a negative history or risk factor element. Values (n) represents cases where answers were available in both CHT and EHR. The agreement is presented as inter-rater reliability (with 95% CI). * Denotes a significant difference between groups. EHR: Electronic health record. CHT: Computerised history taking. CAD: Coronary artery disease. CI: Confidence interval. Obesity: BMI>30 kg/m2 based on self-reported weight and height.

Supplementary Figure 1A. Overview of questions in the computerised history-taking interview relevant for chest pain relief by rest.

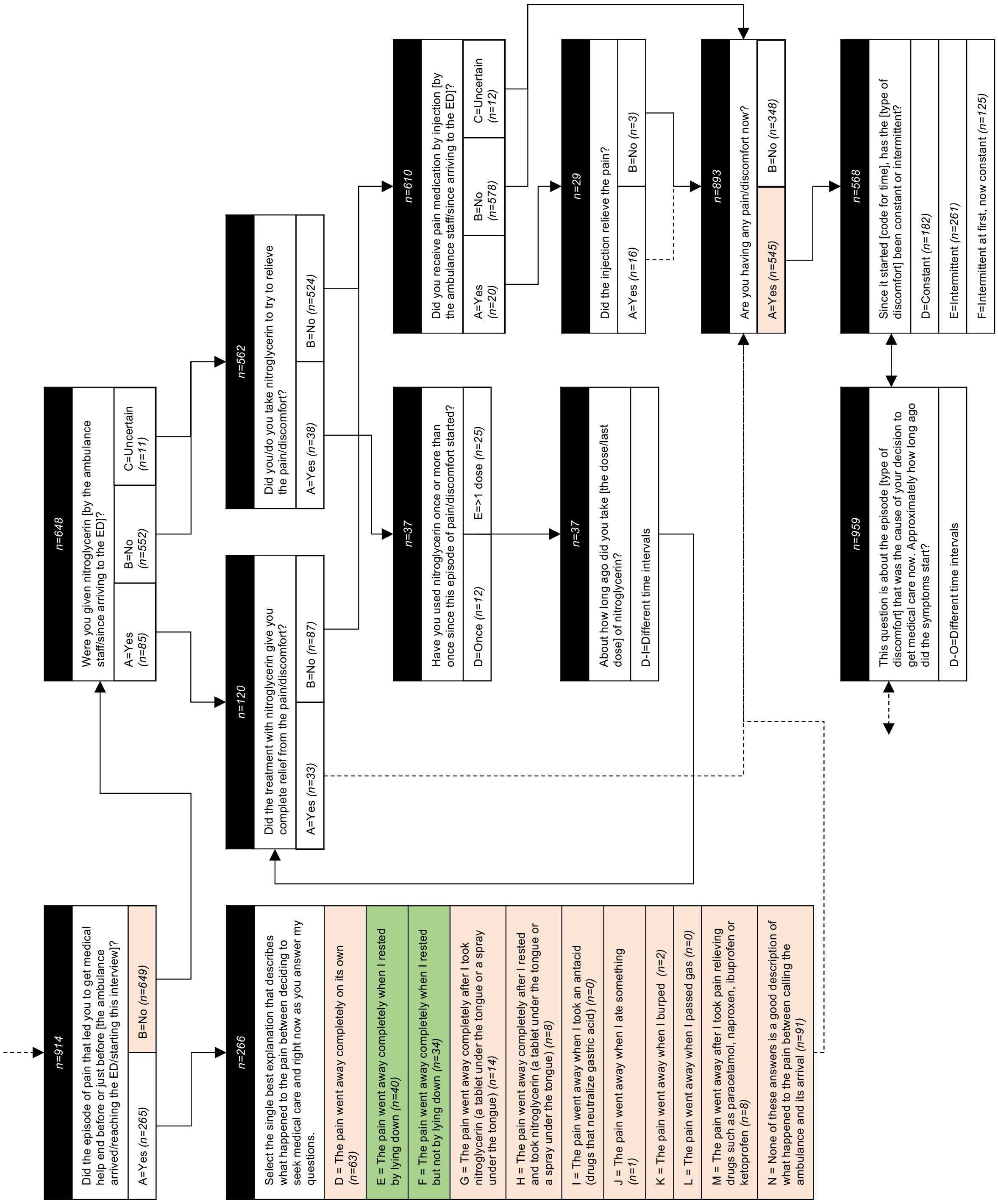

Green and red colours indicate answers generating a positive and negative entry, respectively.

Supplementary Figure 1B. Overview of questions in the computerised history-taking interview relevant for chest pain relief by rest.

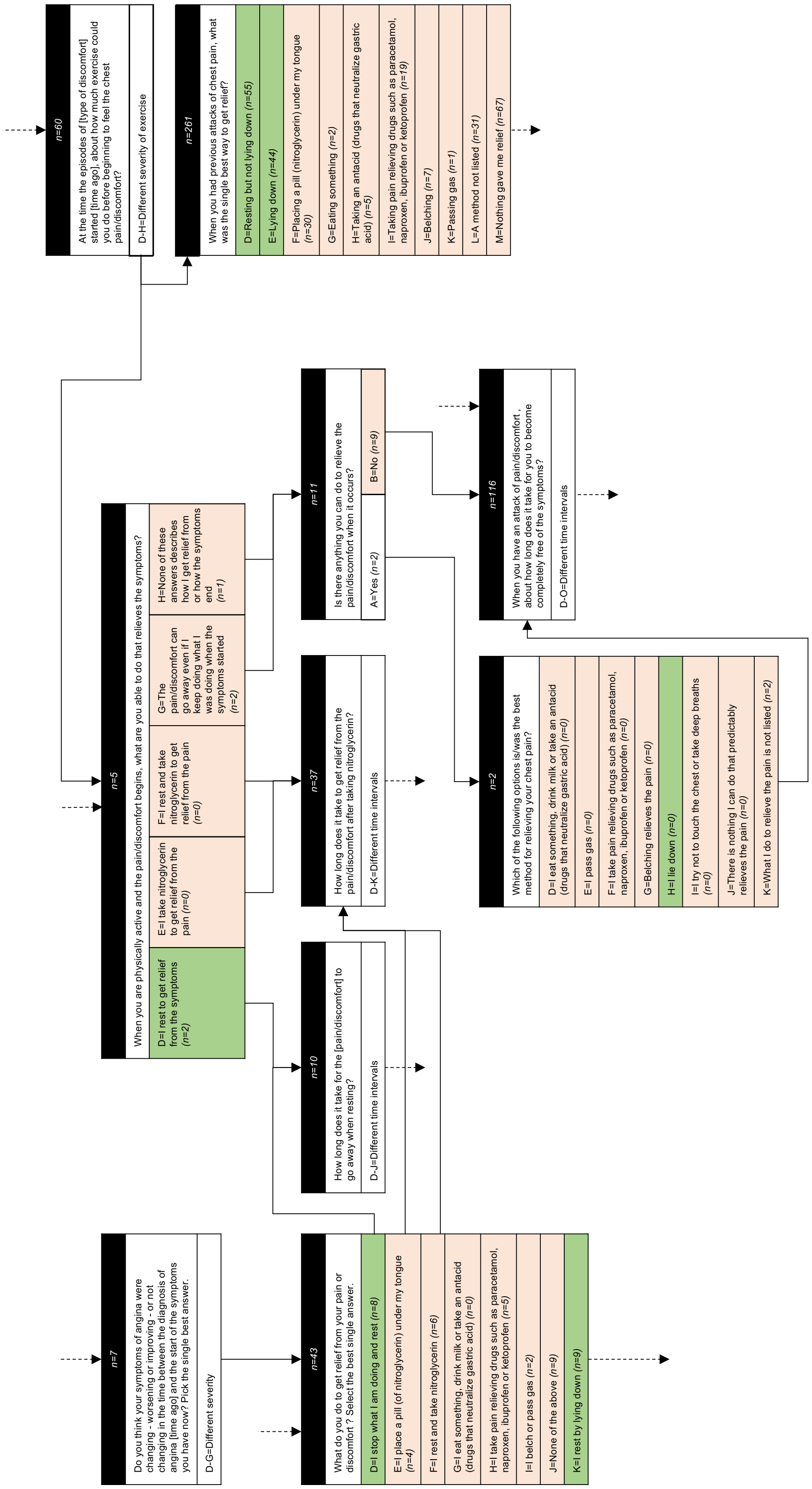

Green and red colour indicate answers generating a positive and negative entry, respectively.

Supplementary Figure 2A. Overview of questions in the computerised history-taking interview relevant for chest pain relief by nitrates.

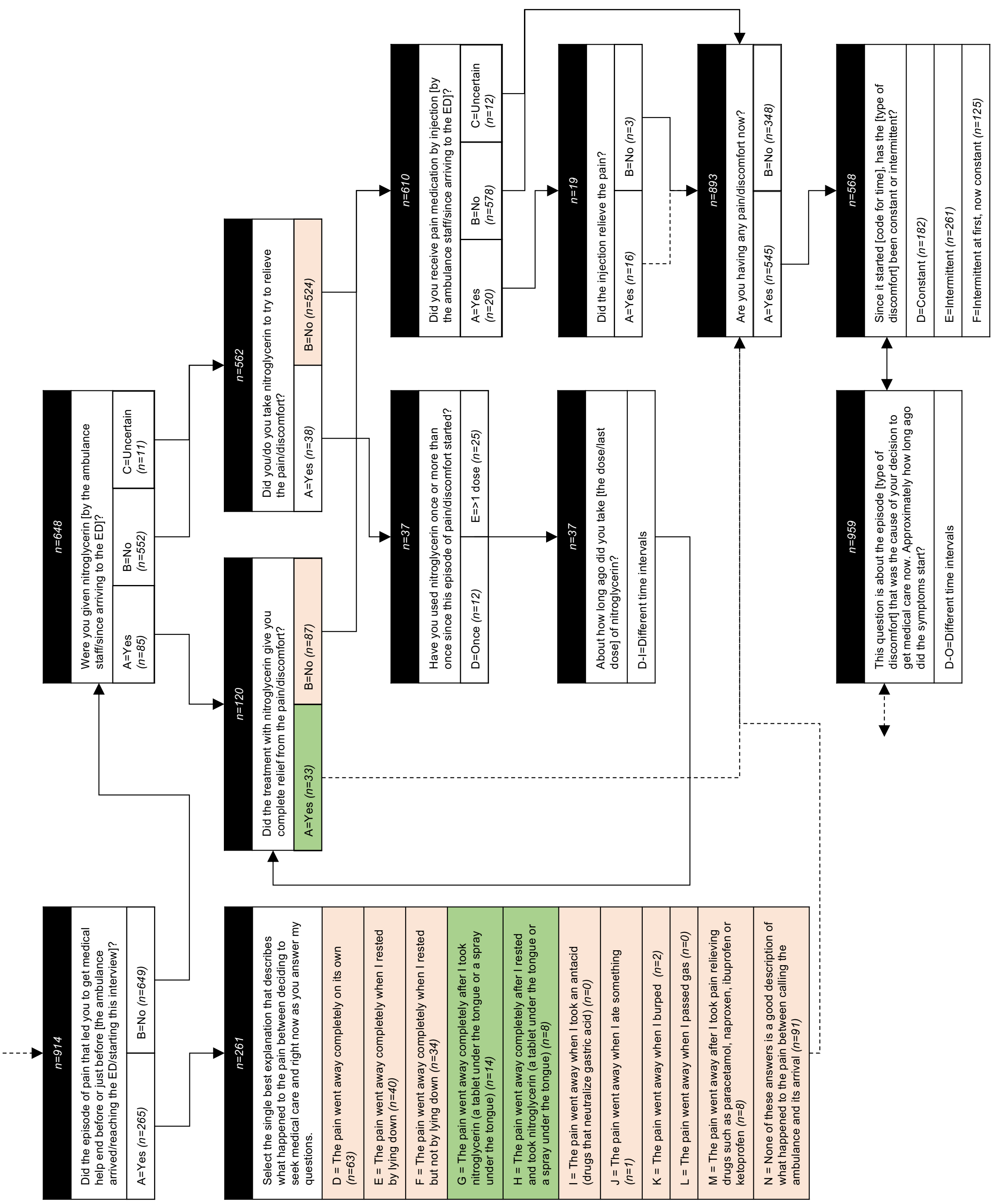

Green and red colour indicate answers generating a positive and negative entry, respectively.

Supplementary Figure 2B. Overview of questions in the computerised history-taking interview relevant for chest pain relief by nitrates.

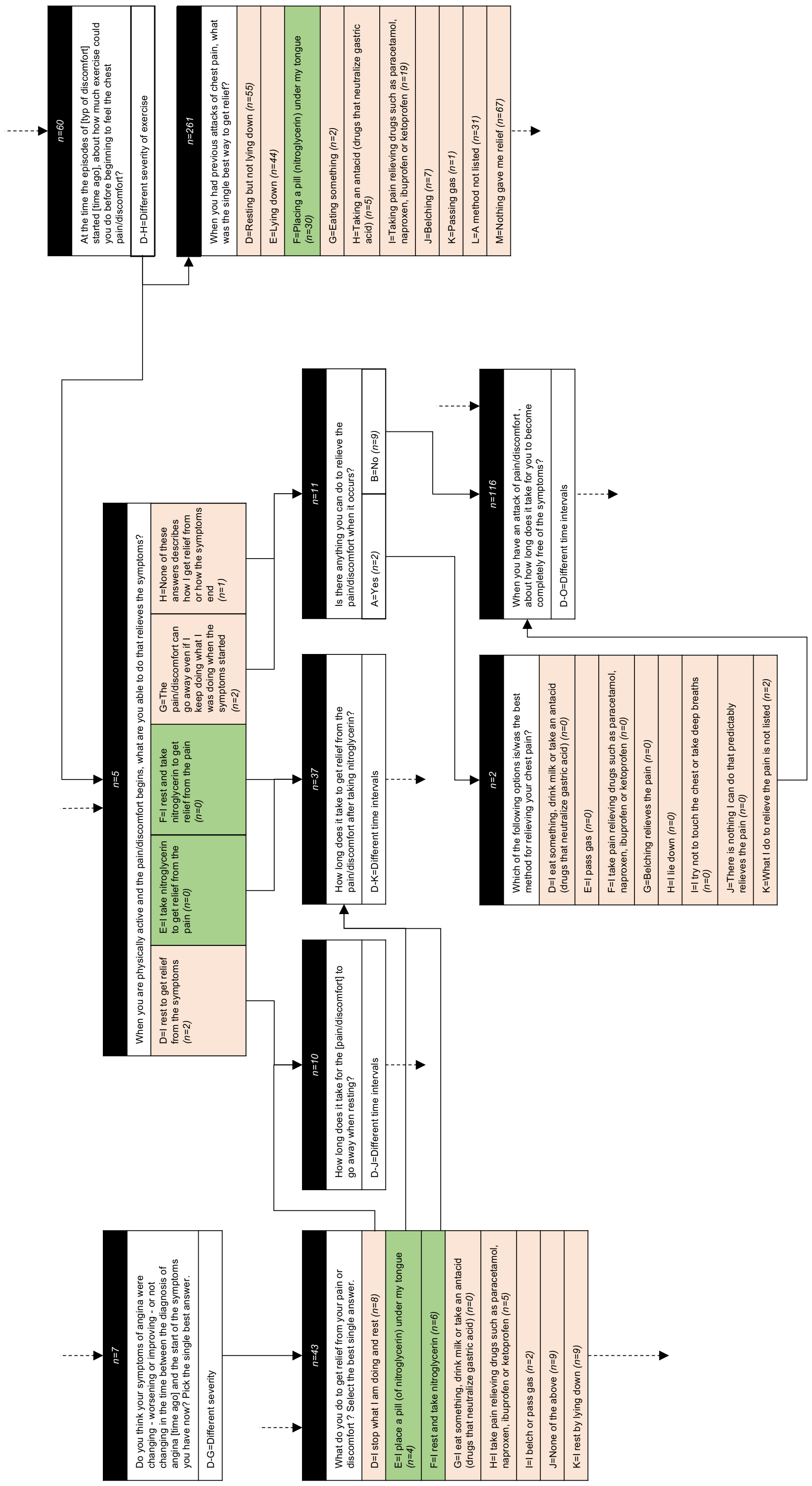

Green and red colour indicate answers generating a positive and negative entry, respectively.

Supplementary Figure 3. Overview of questions in the computerised history-taking interview relevant for worsening/crescendo angina pectoris.

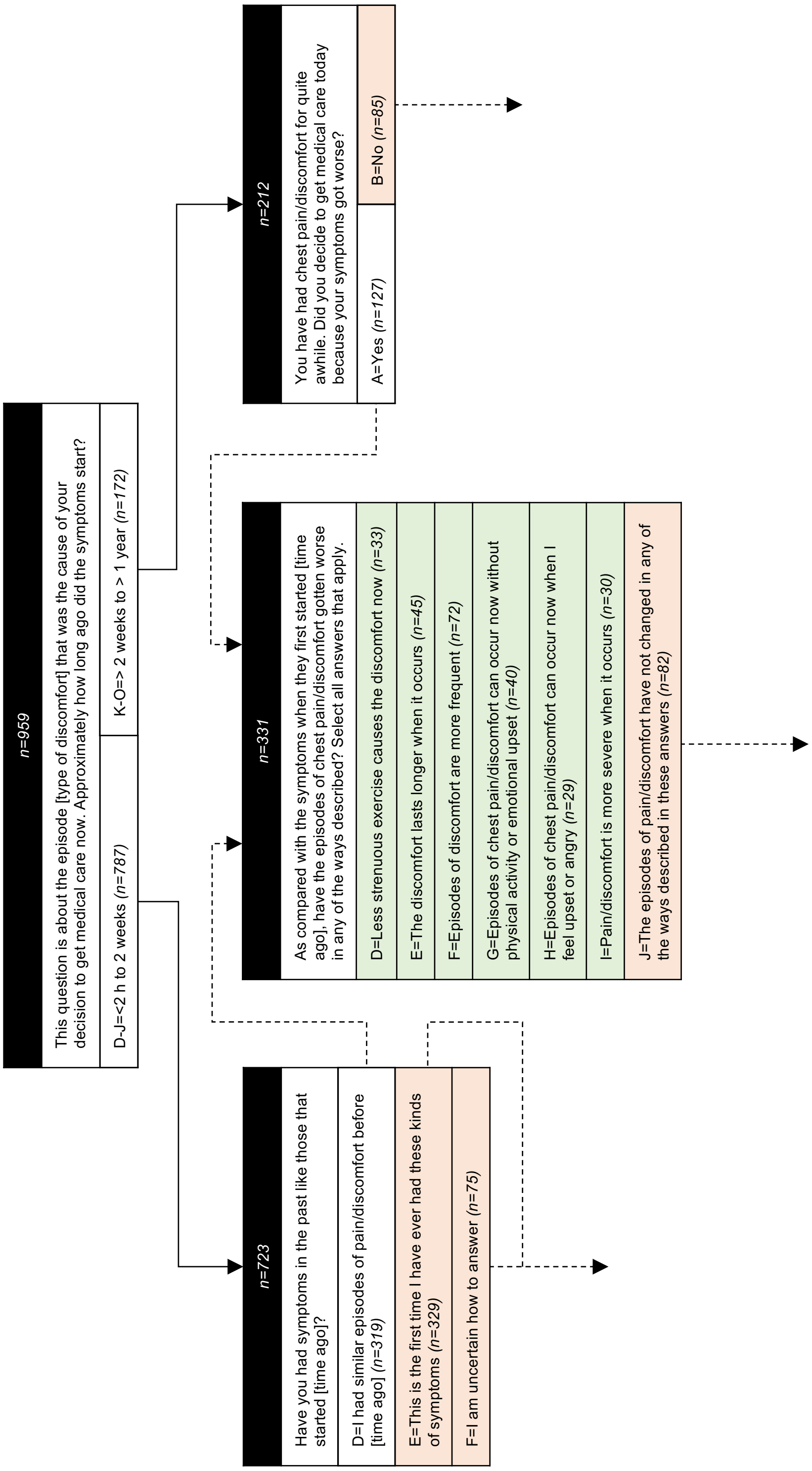

Green and red colour indicate answers generating a positive and negative entry, respectively.
